# Supplementary material for: Modeling glioblastoma heterogeneity as a dynamic network of cell states
Source: Mol Syst Biol. 2021 Sep 16;17(9):e10105. doi: 10.15252/msb.202010105 (PMC8444284; doi:10.15252/msb.202010105)
Supplement: Supplementary file 6 — Source Data for Figure 5 [file MSB-17-e10105-s004.zip › Figure5A_sourcedata/GSEA_3071/hallmarks_stateIV.GseaPreranked.1623742551702/HALLMARK_MYC_TARGETS_V2.html]

Details for gene set HALLMARK\_MYC\_TARGETS\_V2[GSEA]

|  || Dataset | state53071 |
| Phenotype | NoPhenotypeAvailable |
| Upregulated in class | na\_neg |
| GeneSet | HALLMARK\_MYC\_TARGETS\_V2 |
| Enrichment Score (ES) | -0.53387785 |
| Normalized Enrichment Score (NES) | -2.191531 |
| Nominal p-value | 0.0 |
| FDR q-value | 0.0 |
| FWER p-Value | 0.0 |
Table: GSEA Results Summary

  

Fig 1: Enrichment plot: HALLMARK\_MYC\_TARGETS\_V2      
 Profile of the Running ES Score & Positions of GeneSet Members on the Rank Ordered List

  

| PROBE | GENE SYMBOL | GENE\_TITLE | RANK IN GENE LIST | RANK METRIC SCORE | RUNNING ES | CORE ENRICHMENT || 1 | DDX18 |  |  | 2359 | -0.254 | -0.4352 | No |
| 2 | PRMT3 |  |  | 2709 | -0.295 | -0.4859 | No |
| 3 | NOP2 |  |  | 2746 | -0.300 | -0.4767 | No |
| 4 | PPRC1 |  |  | 3040 | -0.338 | -0.5144 | Yes |
| 5 | PHB |  |  | 3089 | -0.343 | -0.5051 | Yes |
| 6 | BYSL |  |  | 3241 | -0.367 | -0.5143 | Yes |
| 7 | MYBBP1A |  |  | 3272 | -0.371 | -0.5002 | Yes |
| 8 | NOC4L |  |  | 3284 | -0.373 | -0.4823 | Yes |
| 9 | IPO4 |  |  | 3340 | -0.380 | -0.4725 | Yes |
| 10 | DCTPP1 |  |  | 3414 | -0.393 | -0.4654 | Yes |
| 11 | AIMP2 |  |  | 3474 | -0.404 | -0.4550 | Yes |
| 12 | TBRG4 |  |  | 3475 | -0.405 | -0.4334 | Yes |
| 13 | WDR74 |  |  | 3479 | -0.405 | -0.4123 | Yes |
| 14 | NDUFAF4 |  |  | 3550 | -0.419 | -0.4032 | Yes |
| 15 | PUS1 |  |  | 3574 | -0.423 | -0.3850 | Yes |
| 16 | RABEPK |  |  | 3625 | -0.432 | -0.3714 | Yes |
| 17 | NOP16 |  |  | 3705 | -0.447 | -0.3625 | Yes |
| 18 | NOLC1 |  |  | 3710 | -0.448 | -0.3393 | Yes |
| 19 | PLK4 |  |  | 3791 | -0.465 | -0.3297 | Yes |
| 20 | HSPD1 |  |  | 3842 | -0.474 | -0.3139 | Yes |
| 21 | PES1 |  |  | 3886 | -0.484 | -0.2962 | Yes |
| 22 | NIP7 |  |  | 3895 | -0.486 | -0.2718 | Yes |
| 23 | WDR43 |  |  | 4202 | -0.568 | -0.2996 | Yes |
| 24 | PLK1 |  |  | 4205 | -0.569 | -0.2696 | Yes |
| 25 | TCOF1 |  |  | 4231 | -0.575 | -0.2436 | Yes |
| 26 | IMP4 |  |  | 4236 | -0.576 | -0.2136 | Yes |
| 27 | SRM |  |  | 4455 | -0.656 | -0.2200 | Yes |
| 28 | PA2G4 |  |  | 4497 | -0.678 | -0.1916 | Yes |
| 29 | NOP56 |  |  | 4556 | -0.708 | -0.1648 | Yes |
| 30 | MRTO4 |  |  | 4716 | -0.806 | -0.1520 | Yes |
| 31 | MCM5 |  |  | 4792 | -0.853 | -0.1207 | Yes |
| 32 | CDK4 |  |  | 4810 | -0.874 | -0.0772 | Yes |
| 33 | MYC |  |  | 4883 | -0.947 | -0.0403 | Yes |
| 34 | MCM4 |  |  | 4947 | -1.011 | 0.0018 | Yes |
| 35 | NPM1 |  |  | 5048 | -1.184 | 0.0460 | Yes |
Table: GSEA details [plain text format]

  

Fig 2: HALLMARK\_MYC\_TARGETS\_V2: Random ES distribution      
 Gene set null distribution of ES for **HALLMARK\_MYC\_TARGETS\_V2**
